# Influence of Actual Curing Conditions on Mechanical Properties of Concrete in Bridge Superstructures

**DOI:** 10.3390/ma16010054

**Published:** 2022-12-21

**Authors:** Jakub Krząkała, Piotr Łaziński, Michael Gerges, Łukasz Pyrzowski, Grzegorz Grządziela

**Affiliations:** 1 Faculty of Civil Engineering, Silesian University of Technology, 44-100 Gliwice, Poland; 2 Faculty of Science & Engineering, University of Wolverhampton, Wolverhampton WV1 1LY, UK; 3 Faculty of Civil and Environmental Engineering, Gdansk University of Technology, 80-233 Gdańsk, Poland; 4 TPA Sp. z o.o., 05-800 Pruszków, Poland

**Keywords:** concrete, prestressed bridge, mechanical properties, curing conditions, rock aggregates

## Abstract

This article presents the research on the mechanical characteristics of concrete in the construction of three concrete bridges. A system of recording the internal temperature of concrete and automatic control of laboratory ovens was used for specimen curing. This allowed the specimens to be cured under conditions similar to those occurring in the structure. Before the construction, reference blocks were used to define similar curing conditions. Maximum setting temperatures ranged from 47.6 °C to 62.0 °C and had a favorable effect on the properties of the concrete at an early age. For concretes with the use of CEM I cement, after 3 days of curing, the strength obtained was up to 8.2 MPa (23%) higher than that for specimens cured under standard conditions. The modulus of elasticity was higher up to 4.9 GPa (21%). For concrete with the use of CEM III cement, these differences were 26.9 MPa (174%) and 10.3 GPa (64%), respectively. After 7 days of curing, the results were close to each other and after 14, 28, and 56 days, higher values were obtained for specimens cured under standard conditions. The value of the modulus of elasticity of concrete was determined using the direct method according to Eurocode and the standard A method. A test load of the bridge was carried out to verify the modulus values obtained from laboratory tests. The highest consistency (99%) between the theoretical deflections and those measured in the test load was achieved when using the stabilized modulus values obtained on specimens cured under structure conditions in the FEM model. The research confirms the necessity of determining the mechanical characteristics of concrete with taking into account the curing conditions of concrete in the structure. A procedure for determining the mechanical properties of concrete for the correct construction of a bridge is proposed. These results can also be used in the development of a digital twin for bridge management.

## 1. Introduction

In the construction process of prestressed concrete bridges, time is an important factor in determining the final cost of construction. In order to reduce the construction time, contractors are pushing to prestress the superstructure as quickly as possible in order to disassemble the formwork or to proceed to the next segment in the case of incremental launching method or balanced cantilever method [1]. To minimize risks in the construction process, it is necessary to know the actual properties of concrete at its early age [2,3]. Portland cement used in massive girders releases heat from hydration during concrete pouring. Inside a typical prestressed bridge beam, an increase in temperature of up to 80 °C is observed after about 24 h [4,5]. This phenomenon has a positive effect on the early strength of concrete [3,6,7,8].

Concrete as a building material is characterized by a very wide variation in deformability [9,10]. The main factors affecting the mechanical characteristics of concrete are w/c ratio, type and amount of aggregate, age of the concrete, and curing conditions of concrete [6,10,11,12]. Research [13] has shown that for normal strength concretes, the compressive strength depends on the contact zone between the aggregate grains and the cement matrix, not on the type of aggregate, whereas the instantaneous modulus of elasticity of concrete depends mainly on the deformability properties of the aggregate used, which differ significantly depending on the type of aggregate [10,14,15,16,17]. Studies [18,19] have shown that aggregate grain size produces no effect.

**Figure 1:**
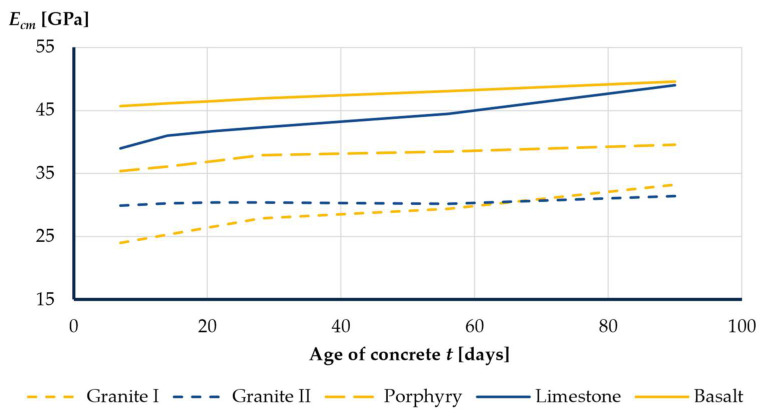
The modulus of elasticity of concrete with the use of various aggregates over time.

In Poland, the bridge constructors commonly use granite, granodiorite, porphyry, limestone, dolomite, amphibolite, and basalt aggregates extracted from many mines across the country. The tests carried out by the authors clearly present a wide variation in the modulus of elasticity of concrete depending on the type of aggregate used (Figure 1). The values ranged from 25 GPa for granite aggregates to 48 GPa for basalt aggregates. Similar results have been reported in the papers [13,16,17,20]. Such differences in deformability require updating the technological design of the bridge.

Laboratory tests of concrete for the construction of bridges have to be performed on specimens cured in water at a controlled temperature of 20 °C [21,22,23]. Most studies on the mechanical properties of concrete perform laboratory tests on specimens cured in water [2,11,13,16,17,18,19]. Studies on the effect of concrete curing conditions [3,7,8,12] on concrete properties do not take into account the variability of conditions over time and the effect of bridge girder massiveness on the exchange of temperature and moisture with the environment.

**Figure 2:**
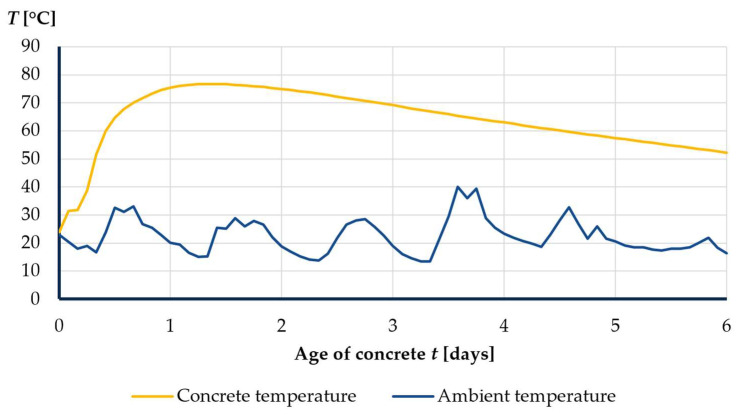
Temperature of concrete inside the superstructure in the first few days.

These curing conditions in the laboratory do not reflect reality. Firstly, the concrete in the superstructure, unlike the specimens in the laboratory, does not have access to an unlimited amount of water. At the construction site, there is only water in the amount specified in the concrete mix recipe. Secondly, the temperature of the concrete in the superstructure during the setting process varies over time and far exceeds the 20 °C that occurs in the laboratory [4,5]. The authors’ own research showed that in the summer months, the temperature inside the girder can reach up to 80 °C (Figure 2). The research presented in this article attempts to take into account the actual curing conditions occurring in the superstructure at the construction site and examine their effect on the mechanical properties of concrete.

Tests of the modulus of elasticity of concrete are performed according to the standard (method A or B) [24]. In the cyclic loading and unloading specimens, the initial plastic deformations are eliminated. In such tests, the values of the modulus of elasticity of concrete differ from the deformability of the structure at the moment of prestressing the span. The prestressing force creates the first stresses in the concrete and the initial plastic deformation occurs. The influence of the curing temperature and the type of aggregate on the mechanical properties of concrete requires the implementation of individual test procedures to obtain the actual values of the properties.

The article presents the results of the research program identifying the mechanical properties of concrete on the example of three bridge structures recently built in Southern Poland: bridge MG-1 over the Oder River, viaduct WG-4 at the grade-separated junction along the provincial road that serves as a bypass for the city of Racibórz and bridge MD-1 over the Kłodnica River along the national road that serves as a bypass for the city of Kędzierzyn-Koźle. The aim of the research was to compare the results of tests on the mechanical properties of concrete carried out using different methods on specimens cured under standard and structure conditions, with the use of a system that allows concrete curing conditions in the superstructure to be replicated in the laboratory. In the case of the WG-4 viaduct, the laboratory test results were verified by in situ tests on its superstructure. Throughout the construction process, the displacements of the measuring points located on the superstructure were measured. The second stage of verification consisted in measuring the span deflections under test loads. The applied numerical models were used to analyze the deformability of the span [25].

## 2. System of Structure Conditions of Concrete Curing

The purpose of the system is to replicate in the laboratory the actual concrete curing conditions occurring in the superstructure at the construction site, so that the test specimens are an authoritative representation of the material used in construction. These replicated conditions have been named structure conditions.

**Figure 3:**
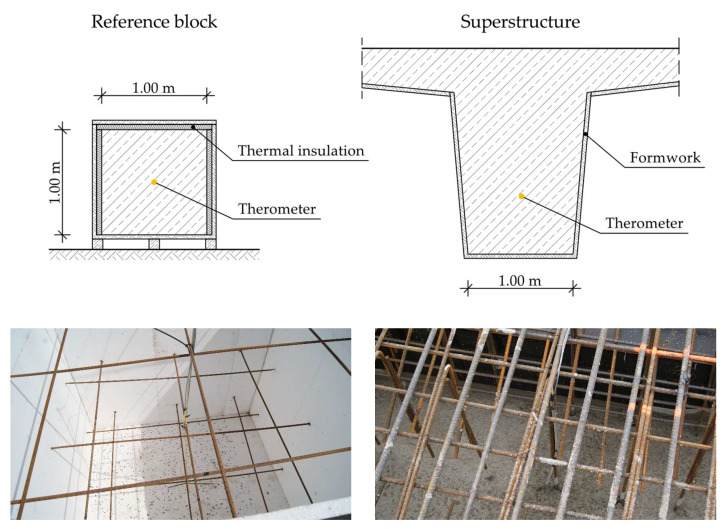
Arrangement of temperature sensors in the reference block and in the girder.

The system consists of temperature sensors placed in the structure, the LB-480 recorder, the LBX server, and a laboratory oven. The temperature sensors are located inside the girder, which is the main superstructure element for the stiffness of the bridge. In the case of preliminary research, before construction begins, a reference block with dimensions similar to the girder is used (Figure 3).

**Figure 4:**
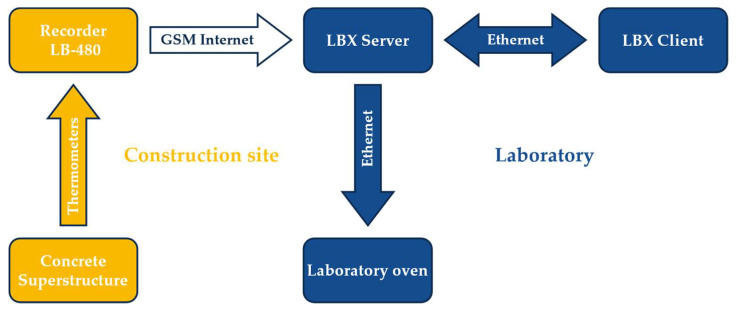
System of structure conditions of concrete curing.

These sensors are connected via cables to a LB-480 recorder located at the construction site. The frequency of measurement is possible to determine; in the case of the described research, a measurement was taken once every 30 min. The recorder is connected to the GSM internet, and on its use sends data to the LBX server located in the laboratory. The laboratory oven is connected to the server, which automatically controls its settings according to the measurements at the construction site. All data and settings can be viewed and possibly changed by the LBX Client, which is also connected to the server. A diagram of the system is shown in Figure 4.

**Figure 5:**
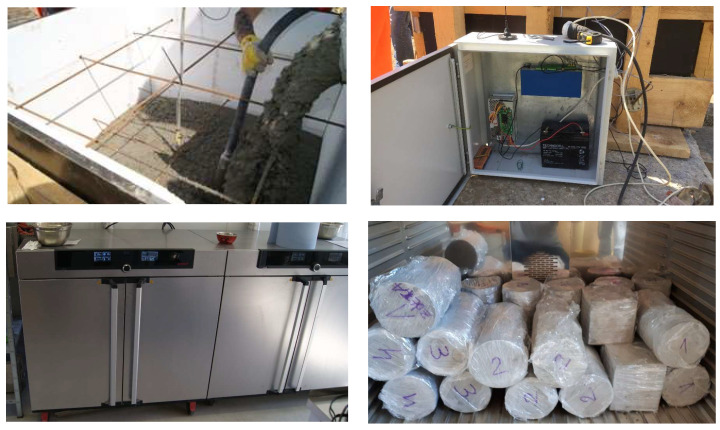
Elements of the system of structure conditions of concrete curing.

In the superstructure, the exchange of water with the environment only occurs over a small area, so it is not an intensive process. To prevent intensive evaporation of water from the specimens in the laboratory oven, they are wrapped in stretch foil after demolding (Figure 5).

## 3. Materials and Methods

### 3.1. Materials

The research was performed on the construction of three bridges. Three different concrete mixes were used. They all consist of CEM I cement, because it is forbidden in Poland to use cements other than CEM I for prestressed bridge structures. For the purpose of this research, a trial mix with the use of CEM III cement was prepared. The mix recipes are presented in Table 1.

The concrete pouring of the test blocks took place at different times of the year. The different weather conditions had a significant impact on the temperature of the concrete mixture at the construction site and the maximum temperature of the concrete during the setting process (Table 2).

### 3.2. Methods

The mechanical properties of concrete were determined on the basis of destructive tests of specimens cured under standard and structure conditions. The first part of the research is the preliminary research that takes place before construction begins. After the concrete mix recipe is approved, a reference block is made. The second part of the research is the tests carried out on the concrete used for the construction of the bridge.

**Figure 6:**
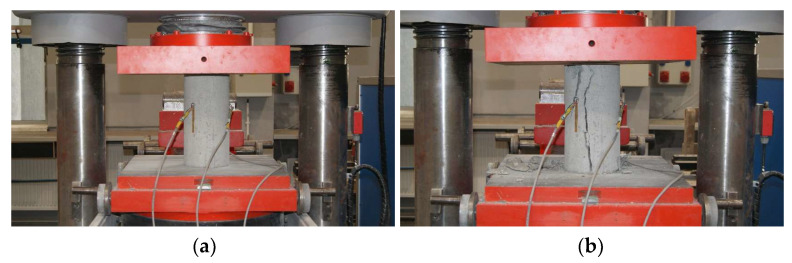
Concrete specimen at the time of testing: prepared for test (**a**) and after destruction (**b**).

For the analysis of the concrete mechanical properties, a minimum of 24 cylindrical specimens with a diameter of *d* = 150 mm and a height of *h* = 300 mm were prepared [23]. Half of them were cured under standard conditions in water, while the other half under structure conditions in the laboratory oven. The tests of the modulus of elasticity of concrete were carried out on a class 1 compression testing machine [26]. The change in stress in the concrete was recorded with sampling every 0.2 s. Deformation measurement was carried out along three lines arranged on the sidewall every 120° [24] with sampling as for stress recording (Figure 6).

The analysis of the concrete modulus of elasticity was carried out using two methods:

- direct method;
- method A.

Direct method is proposed by the authors as an exact interpretation of the definition of the modulus of elasticity of concrete described in the Eurocode [27]. The specimens are subjected to a single loading cycle until failure (Figure 6). For the calculation, the formulas used were

(1) $$E_{cm\;}=\frac{{0.4\;f}_{cm}}{\Delta \varepsilon },\;$$

(2) $$\Delta \varepsilon =\frac{\Delta l}{l_{0}},\;$$

where

*f_cm_*—compressive strength of concrete;
*Δε*—strain increase in the concrete induced by the stress of 0.4 *f_cm_*;
*Δl*—change in specimen length due to stress of 0.4 *f_cm_*;
*l*_0_—base length of concrete specimen.

**Figure 7:**
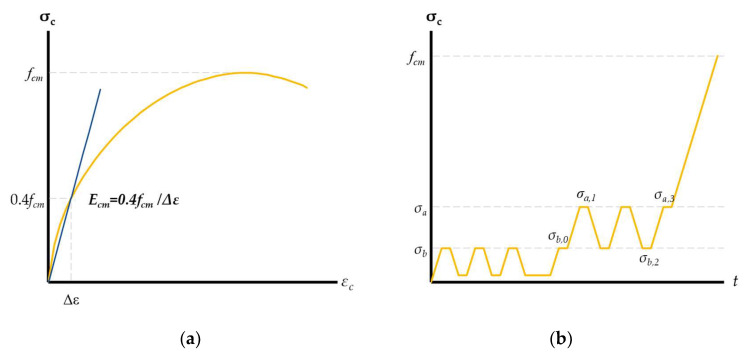
Specimen loading process: direct method (**a**) and method A (**b**).

Method A, according to the standard [24], was used to determine two values for the secant modulus of elasticity: the initial modulus *E_c,o_* and stabilized modulus *E_c,s_*. The specimens are subjected to cyclic loading and unloading, as shown in Figure 7. For the calculation, the formulas used were

(3) $$E_{c,0}=\frac{\sigma_{a,1}{\;-\;\sigma }_{b,0}}{\varepsilon_{a,1}{\;-\;\varepsilon }_{b,0}},$$

(4) $$E_{c,s}=\frac{\sigma_{a,3}{\;-\;\sigma }_{b,2}}{\varepsilon_{a,3}{\;-\;\varepsilon }_{b,2}},\;$$

where

*σ_a,i_*—registered stresses at the level of 1/3 *f_cm_*;
*σ_b,i_*—registered stresses at the level of 0.15 *f_cm_*;
*ε_a,i_*—registered strain of the concrete induced by the stress of *σ_a,I_*;
*ε_b,i_*—registered strain of the concrete induced by the stress of *σ_b,i_*.

## 4. Results

### 4.1. Preliminary Research

In each of the three bridges, preliminary research using reference blocks was carried out before construction started. The concrete was made following the approved mix recipes. As a result, the actual mechanical properties of concrete as a function of age were determined. The obtained values of compressive strength and modulus of elasticity allowed to update the technological design. The preliminary tests were used to update the pre-camber and the prestressing program.

**Figure 8:**
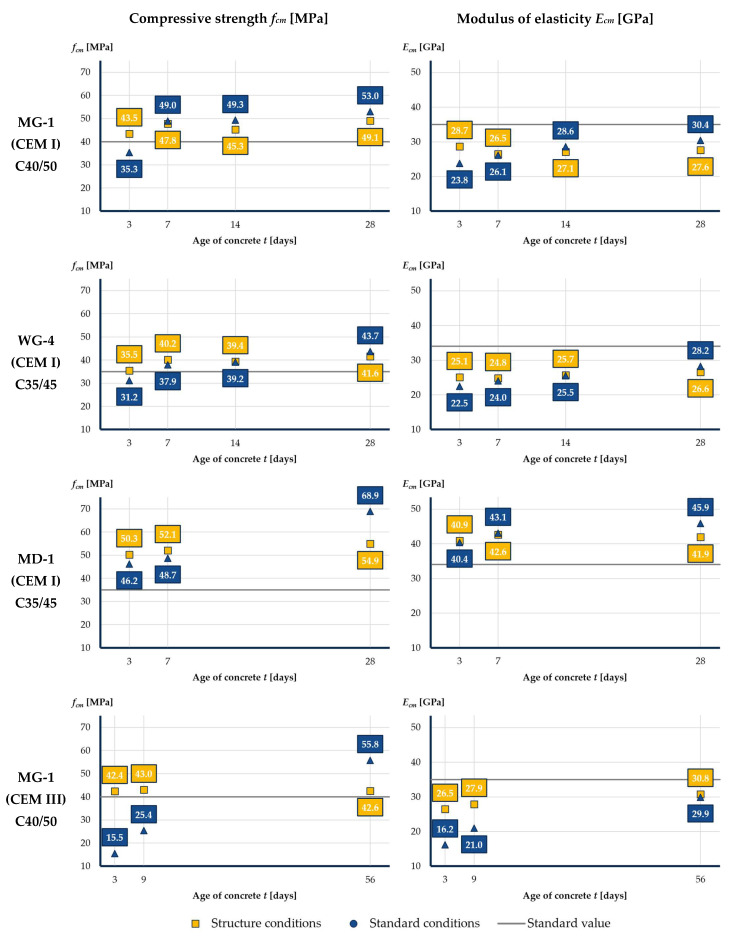
Compressive strength and modulus of elasticity of concrete over time, taking into account curing conditions.

The results of the laboratory tests are summarized in Table 3 and Figure 8. After 3 days of curing, higher values of compressive strength and modulus of elasticity were obtained for the specimens cured under the structure conditions. The difference in results is significant especially for concrete with the use of CEM III cement. This indicates the positive effect of the high curing temperature on the early increase in mechanical properties of concrete.

After 7 days of curing, the results of the concrete properties were similar for both types of specimens. In the following days, higher values of concrete properties were observed for specimens cured under standard conditions. The results indicate that the increase in the mechanical properties of concrete over time does not occur in the structure. Such differences in the results obtained depending on the way in which the specimens were cured confirm the need to take into account the curing conditions of the concrete in the structure in the laboratory tests.

In addition, the research has proven that the actual mechanical properties of concrete with the use of CEM III cement used are characterized by a rapid increase in the first few days, similarly to concrete with the use of CEM I cement.

The results confirm the relatively low value of modulus of elasticity of concrete with the use of granite aggregate and the relatively high value for concrete with the use of basalt aggregate.

### 4.2. Research on the Bridge Structure

#### 4.2.1. Laboratory Tests

Concreting of the superstructure of the WG-4 bridge took place on November 26th, 2020. A mix of S3 consistency, air content 3.8%, and temperature of 12.3 °C was poured. The maximum temperature of 45.8 °C was reached 32 h after concreting the superstructure. During this research, the ambient temperature was approximately 0 °C ± 5 °C.

The tests were carried out after 6 days of curing at the moment of prestressing the superstructure and additionally after 28 days. In both cases, the specimens were tested with the use of the direct method and the method A.

The results of the laboratory tests are summarized in Table 4. The tests confirmed the conclusions of the preliminary research. For each method, the results were higher for specimens cured under structure conditions at an early age and for specimens cured under standard conditions at a later stage.

The results of the modulus of elasticity tests using the direct method are close to the initial modulus values from the tests carried out using method A (including initial plastic deformation). The stabilized modulus values, as expected, are higher.

#### 4.2.2. Test Load

**Figure 9:**
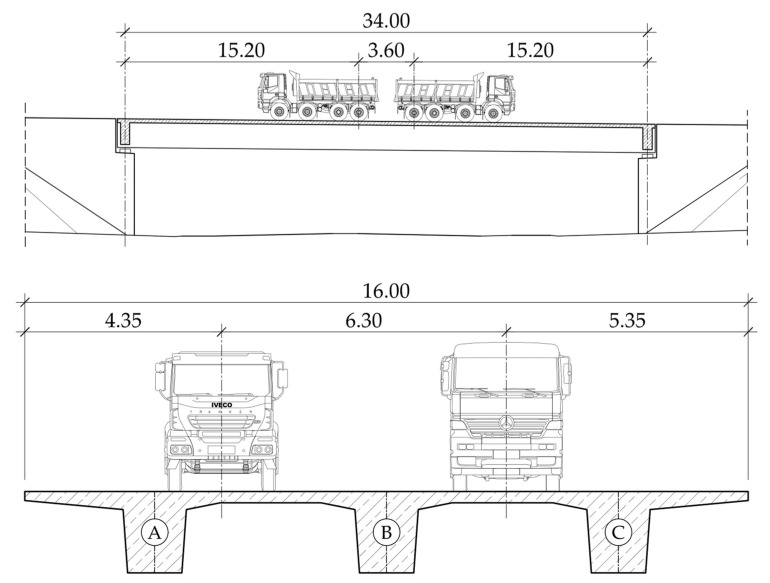
Truck location on the superstructure.

On 29 April 2021, 154 days from the concreting of the superstructure, the vertical displacements were measured under a static test load. The load of the bridge was four four-axle trucks with a total weight of 129.04 tons. The trucks were placed on the bridge in order to cause the maximum vertical displacement of the superstructure (Figure 9).

**Figure 10:**
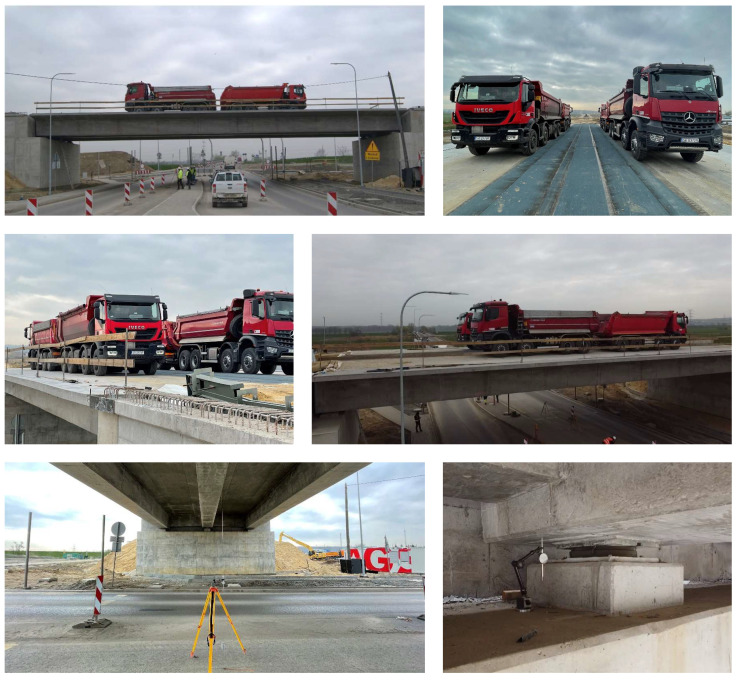
Superstructure of WG-4 bridge subjected to test load.

Once the trucks were placed on the bridge, measurements of the displacements of both the superstructure and the supports were conducted. Measurements were taken every 10 min until the displacements were stabilized. The test was carried out before laying the wearing surface and utilities placement, which allowed to eliminate its influence on the stiffness of the superstructure (Figure 10).

**Figure 11:**
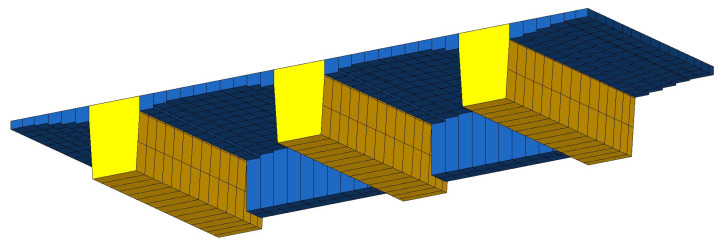
Division of the model into finite elements: beams (yellow) and plates (blue).

Numerical analysis of the superstructure was performed using the FEM program. The main girders were modeled using beam elements, while the deck slab and cross beams were modeled using shell elements (Figure 11).

**Figure 12:**
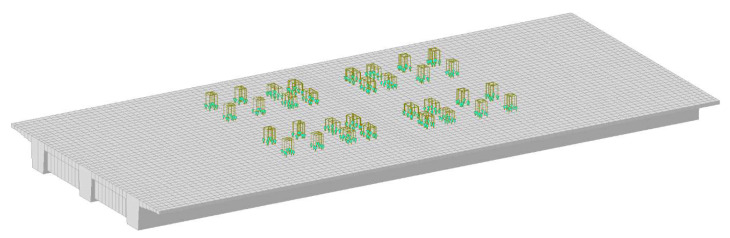
Numerical model of WG-4 superstructure with truck load.

Numerical analyses were performed using different values of the modulus of elasticity: the standard value and those obtained from laboratory tests. The load of the trucks was modeled as pressure on the wheel-structure contact surface (Figure 12).

Table 5 presents the results of the test load with a comparison to the results from the numerical analyses. For the standard modulus of elasticity (*E_cm_* = 34.0 GPa), higher deflections were obtained than the values measured in the in situ tests. For the modulus of elasticity determined by the laboratory method A on specimens cured under standard conditions (*E_cm_* = 42.9 GPa), the theoretical deflections obtained were smaller than the values from in situ tests. A high level of consistency in the results was achieved when the stabilized modulus value from method A obtained for specimens cured under structure conditions (*E_cm_* = 38.6 GPa) was used in the numerical model. The deflection ratio ranged from 97.6% to 100.5%.

These values of deflection ratio indicate that concrete in an already prestressed structure has a higher value of modulus of elasticity than at the moment of prestressing. Furthermore, such high deflection consistency clearly presents that the results from tests carried out on specimens cured under structure conditions are close to the actual properties of the material built into the superstructure. The comparative study has confirmed the elastic work of the superstructure after eliminating the initial plastic deformations that occur at the moment of prestressing. Taking into account the prestressing phase and the service phase of prestressed bridges, it is necessary to determine two values of the modulus:

- initial value determined with the direct method (with initial plastic deformation)—prestressing phase;
- final value determined with method A (without initial plastic deformation)—useable phase.

## 5. Discussion

The purpose of the research was to propose a procedure for concrete testing that would provide results that would allow a higher consistency between the deflections of the bridge spans measured during the test load and those obtained from the numerical model. The research used a system of recording the internal temperature of the concrete and automatic control of laboratory ovens, which made it possible to cure the specimens under conditions similar to those occurring in the superstructure. The results obtained from the laboratory tests were compared with the results of the test load of the bridge.

A comparative study of the mechanical properties of concrete confirmed the beneficial effect of high concrete curing temperature on the early compressive strength and modulus of elasticity of concrete. Similar conclusions were reached in the studies [3,7,8,12]. This phenomenon can be referenced with the use of a monitoring system for the curing conditions of concrete in the superstructure. For the purposes of deciding on the timing of prestressing at an early age of concrete, the results of tests carried out on specimens cured under structure conditions should be considered authoritative. This minimizes the risks associated with decision-making at the construction site and reduces the construction time, thus reducing construction costs. The monitoring system implemented allows control of the phenomena occurring in the structure, which vary according to the massiveness of the girders and the time of concreting. This effect was not considered in the laboratory tests in the studies [3,7,8,19].

A crucial issue in the construction process of prestressed bridges is the deformability of the concrete. This can vary significantly depending on the type of aggregate used [9,10,11,16,17]. The actual value of the modulus of elasticity of the concrete influences the correct prestressing program and the correct values of the pre-camber. For this purpose, it is necessary to determine the modulus of elasticity during the approval process of the concrete mix recipe. The presented method of determining the mechanical properties with the use of a reference block takes into account the thermal phenomena occurring during the concrete setting in the massive bridge girder. This approach allows the numerical model to be updated with the actual values of the material properties even before construction starts.

The research confirmed the operation of a prestressed concrete bridge in two phases. The first phase is the prestressing process of the superstructure, in which the initial plastic deformations occur. In this phase, the effectiveness of determining the modulus of elasticity of concrete using the direct method on specimens cured under structure conditions was confirmed. This approach resulted in a structure geometry that was consistent to within 1 cm of the designed grade line.

The second phase is the usage phase of the bridge. By performing the test load of the structure without any equipment installed, the deformability of the concrete in the usage phase was determined. On this basis, a reduction in the deformability of the concrete (increase in the value of modulus of elasticity) was confirmed, which is related to the elimination of plastic deformation. The determination of the modulus of elasticity using method A on specimens cured under structure conditions was considered authoritative.

In addition, the results of tests on the mechanical properties of concrete with CEM III cement confirmed a rapid increase in the compressive strength and modulus of elasticity at an early age as in the case of concrete with CEM I cement, which allows the use of such concrete for the construction of prestressed bridges.

## 6. Conclusions

The research confirmed the significant influence of concrete curing conditions on the development of its mechanical properties over time. Tests carried out on specimens cured under the conditions specified by the standard underestimate the values of the compressive strength and modulus of elasticity of the concrete at its early age relative to the actual values and significantly overestimate them in the long term.

For the correct design and construction of the bridge, it is necessary to determine the modulus of elasticity using laboratory tests. The specimens for these tests should be cured under structure conditions close to the actual conditions in the structure, which differ significantly from those specified in the standard. In addition, for prestressed bridges, it is necessary to determine two values of the modulus of elasticity—with and without taking into account the initial plastic deformation.

The results obtained from laboratory tests and verified by the test load should be the base for the creation of the mechanical twin that is part of the digital twin of the bridge [28,29].

Proposed procedure for determining the actual mechanical properties of concrete built into the superstructure of the bridge:

- Tests of concrete specimens with the use of a reference block and monitoring system after approval of the concrete mix recipe;
- Update of the prestressing program and technological design of the superstructure using the modulus of elasticity value *E_cm_* determined with the direct method on specimens cured under structure conditions at the age of scheduled prestressing;
- Deciding on the prestressing of the bridge based on the results of tests of the strength and modulus of elasticity of concrete on specimens cured under structure conditions;
- In the analyses of the usage phase, the use of the stabilized modulus of elasticity of concrete *E_csm_* determined with method A on specimens cured under structure conditions.

## Figures and Tables

**Table 1:** Concrete mix recipes.

	Cement	Sand	Coarse Aggregate	Water	Air Content
MG-1	CEM I 42.5R 380 kg/m^3^	660 kg/m^3^	granite 1130 kg/m^3^	w/c = 0.39 150 kg/m^3^	5.0%
WG-4	CEM I 42.5R 370 kg/m^3^	670 kg/m^3^	granite 1130 kg/m^3^	w/c = 0.41 150 kg/m^3^	5.0%
MD-1	CEM I 42.5R 380 kg/m^3^	683 kg/m^3^	basalt 1251 kg/m^3^	w/c = 0.42 157 kg/m^3^	5.0%
MG-1 (CEM III)	CEM III/A 42.5N 390 kg/m^3^	660 kg/m^3^	granite 1110 kg/m^3^	w/c = 0.38 150 kg/m^3^	5.0%

**Table 2:** Temperatures in reference blocks.

	Date	Consistency Class	Air Content	Mixture Temperature	Ambient Temperature	Max. Concrete Temperature
MG-1	27 IV	S3	5.2%	19.0 °C	15 °C ± 10 °C	62.0 °C
WG-4	10 XI	S3	5.0%	13.3 °C	10 °C ± 5 °C	47.6 °C
MD-1	12 XII	S3	5.3%	20.2 °C	5 °C ± 2 °C	57.6 °C
MG-1 (CEM III)	24 VIII	S3	4.8%	24.5 °C	25 °C ± 10 °C	56.9 °C ^1^

^1^ In comparison, the maximum temperature in segment III of MG-1 bridge structure (concrete with the use of CEM I) concreted at a similar time was 76.7 °C (Figure 2).

**Table 3:** Summary of laboratory test results–preliminary research.

Age of Concrete *t* [Days]		3	7 ^1^/9 ^2^	14	28 ^1^/56 ^2^
**MG-1 bridge structure (CEM I)**					
Compressive strength *f_cm_* [MPa]	Structure conditions	43.5 σ = 0.9	47.8 σ = 1.1	45.3 σ = 1.2	49.1 σ = 0.4
	Standard conditions	35.3 σ = 0.6	49.0 σ = 1.1	49.3 σ = 0.1	53.0 σ = 1.3
Modulus of elasticity direct method *E_cm_* [GPa]	Structure conditions	28.7 σ = 0.6	26.5 σ = 0.2	27.1 σ = 0.2	27.6 σ = 0.3
	Standard conditions	23.8 σ = 0.6	26.1 σ = 0.5	28.6 σ = 0.6	30.4 σ = 0.1
**WG-4 bridge structure (CEM I)**					
Compressive strength *f_cm_* [MPa]	Structure conditions	35.5 σ = 0.9	40.2 σ = 1.0	39.4 σ = 0.8	41.6 σ = 0.4
	Standard conditions	31.2 σ = 0.7	37.9 σ = 1.0	39.2 σ = 0.6	43.7 σ = 1.2
Modulus of elasticity direct method *E_cm_* [GPa]	Structure conditions	25.1 σ = 1.0	24.8 σ = 0.5	25.7 σ = 0.3	26.6 σ = 0.1
	Standard conditions	22.5 σ = 0.4	24.0 σ = 0.6	25.5 σ = 0.1	28.2 σ = 0.9
**MD-1 bridge structure (CEM I)**					
Compressive strength *f_cm_* [MPa]	Structure conditions	50.3 σ = 1.8	52.1 σ = 1.1	-	54.9 σ = 1.2
	Standard conditions	46.2 σ = 0.9	48.7 σ = 0.6	-	68.9 σ = 2.4
Modulus of elasticity direct method *E_cm_* [GPa]	Structure conditions	40.9 σ = 1.6	42.6 σ = 0.7	-	41.9 σ = 0.6
	Standard conditions	40.4 σ = 0.2	43.1 σ = 0.3	-	45.9 σ = 1.1
**MG-1 bridge structure (CEM III)**					
Compressive strength *f_cm_* [MPa]	Structure conditions	42.4 σ = 0.3	43.0 σ = 0.3	-	42.6 σ = 0.2
	Standard conditions	15.5 σ = 0.7	25.4 σ = 0.3	-	55.8 σ = 0.3
Modulus of elasticity direct method *E_cm_* [GPa]	Structure conditions	26.5 σ = 0.3	27.9 σ = 0.3	-	30.8 σ = 0.5
	Standard conditions	16.2 σ = 0.2	21.0 σ = 1.7	-	29.9 σ = 1.4

^1^ A number of 7 and 28 days for concrete with the use of CEM I; ^2^ a number of 9 and 56 days for concrete with the use of CEM III.

**Table 4:** Summary of laboratory test results–WG-4 superstructure.

Age of Concrete *t* [Days]		6	28
Compressive strength *f_cm_* [MPa]	Structure conditions	43.7 σ = 0.5	50.7 σ = 1.0
	Standard conditions	40.5 σ = 0.4	53.3 σ = 0.6
Modulus of elasticity direct method *E_cm_* [GPa]	Structure conditions	27.6 σ = 0.2	30.1 σ = 0.4
	Standard conditions	25.0 σ = 0.3	30.7 σ = 0.5
Modulus of elasticity method A—initial modulus *E_c0m_* [GPa]	Structure conditions	27.4 σ = 0.3	30.3 σ = 0.6
	Standard conditions	26.0 σ = 1.0	30.6 σ = 0.3
Modulus of elasticity method A—stabilized modulus *E_csm_* [GPa]	Structure conditions	34.6 σ = 0.7	38.6 σ = 0.6
	Standard conditions	33.9 σ = 1.2	42.9 σ = 0.5

**Table 5:** Summary of the results of test load and numerical analyses.

		Girder A	Girder B	Girder C
Elastic deflection	*u_e_* [mm]	7.96	7.87	7.11
Theoretical deflection Modulus acc. to Eurocode 2 *E_cm_* = 34.0 GPa	*u_t_*_1_ [mm]	8.99	9.15	8.24
	*u_e_*/*u_t_*_1_	88.5%	86.0%	86.3%
Theoretical deflection Stabilized modulus (method A) Structure conditions *E_csm_* = 38.6 GPa	*u_t_*_2_ [mm]	7.92	8.06	7.26
	*u_e_*/*u_t_*_2_	100.5%	97.6%	97.9%
Theoretical deflection Stabilized modulus (method A) Standard conditions *E_csm_* = 42.9 GPa	*u_t_*_3_ [mm]	7.13	7.25	6.53
	*u_e_*/*u_t_*_3_	111.6%	108.6%	108.9%

## Data Availability

The data presented in this study are available on request from the corresponding author.
